# Prevalence and determinants of multimorbidity in the Canadian population

**DOI:** 10.1371/journal.pone.0297221

**Published:** 2024-01-30

**Authors:** Xiang Xiao, Jeremy Beach, Ambikaipakan Senthilselvan

**Affiliations:** 1 School of Public Health, University of Alberta, Edmonton, Alberta, Canada; 2 Department of Medicine, University of Alberta, Edmonton, Alberta, Canada; IUPUI: Indiana University Purdue University at Indianapolis, UNITED STATES

## Abstract

Multimorbidity, which is defined as having at least two or more chronic diseases concurrently, has been a rising public health issue in recent years in Canada and worldwide. The increasing prevalence of multimorbidity has posed a burden on the current health care system and quality of life for the Canadian population. There is a lack of up-to-date research on determinants of multimorbidity in the Canadian population, which is necessary to better understand and prevent multimorbidity. This study aims to determine the prevalence and risk factors of multimorbidity in the middle-aged and older Canadian adult population. Multivariable logistic regression analyses incorporating survey weights and biologically plausible interactions were conducted to examine the determinants of multimorbidity using data from the 2017/2018 Canadian Community Health Survey (CCHS). Of the 113,290 CCHS participants, 82,508 subjects who were aged 35 years and above were included in the study. The prevalence of multimorbidity was 22.20% (95% CI: 21.74%, 22.67%) and was greater for females. Multimorbidity was more likely in subjects who were obese, abstaining from alcohol, inactive, had a lower education level, widowed, divorced, or separated and was less likely among subjects living in Quebec. The protective effect of household income on multimorbidity decreased with age. Current smokers who reported extreme stress were more likely to have multimorbidity. Multimorbidity is associated with various determinants that need to be considered in chronic disease control and prevention. These results suggest that future research should focus not only on these determinants but also on the relationships between them. A future longitudinal study is required to provide causal evidence for the study findings.

## Introduction

Multimorbidity, which is defined as having at least two or more chronic diseases concurrently, has been a rising public health issue in recent years in Canada and worldwide [1,2]. One in three Canadians has at least one chronic disease [3]. According to Statistics Canada, cardiovascular disease, diabetes, and chronic lower respiratory disease accounted for 25.7% of all-cause deaths in 2018 [4]. Multimorbidity poses a further layer of complexity to the treatment of patients with these conditions. Based on the Canadian Chronic Disease Surveillance System, the prevalence of multimorbidity in middle-aged and older Canadians continued to increase linearly from 20.5% in 2001 to 26.5% in 2011 [5]. The increasing prevalence of multimorbidity has resulted in a burden on the current health care system, as well as on the quantity and quality of life for the Canadian population [6]. Multimorbidity is associated with an increase in hospitalization and health care resource needs, reduced health-related quality of life, and increased health care costs [7,8].

In a systematic review, current known factors associated with multimorbidity were grouped into individual and biomedical factors, socioeconomic characteristics, health behavior and social and environmental factors [9]. A study of older adults showed that higher wealth was associated with lower odds of multimorbidity [10]. In addition, women were found to have a higher risk of multimorbidity than men [11]. Several studies have examined the determinants associated with multimorbidity in the Canadian population [2,12]. However, overall, there appeared to be relatively sparse up-to-date research on determinants of multimorbidity in the Canadian population, which is necessary for a better understanding and prevention of multimorbidity. In addition, while prior research has investigated the risk factors associated with multimorbidity, there exists a need to explore biologically plausible interactions among determinants within individuals with multimorbidity. For example, Barnett and colleagues highlighted differences in changes in the prevalence of multimorbidity with socio-economic disparities among distinct local income clusters in their early study [13]. A more recent longitudinal study that investigated social determinants and the likelihood of multimorbidity, also acknowledged the necessity to incorporate the concept of effect modification noting the influence of social support on perceived stress levels, varied between individuals with differing social status [14]. Therefore, our study aimed to determine the prevalence of multimorbidity in the middle-aged and older Canadian population, and particularly to identify associated determinants using data from a recent Canadian Community Health Survey (CCHS), and after allowing for plausible biological interactions between risk factors in the analysis.

## Materials and methods

### Data source

This study is based on an analysis of the Public Use Microdata File (PUMF) of the Canadian Community Health Survey (CCHS) conducted in 2017/2018, including Canadians aged 35 years and above [15]. The CCHS is a cross-sectional survey that collects information related to health status, health care utilization, and health determinants in the Canadian population aged 12 years and above, living in ten provinces and three territories, excluding people living on the reserves and aboriginal settlements, full-time Canadian forces, the institutionalized population, children living in foster care, and people living in the health regions of Région du Nunavik and Région des Terres-Cries-la-Baie-James in Quebec. A multi-stage sampling allocation strategy was used to provide a representative sampling distribution to the health regions and the provinces. First, the sample was allocated among the provinces based on their respective population sizes. Secondly, the sample was allocated according to the number of people in those health regions. The collection of data was conducted through telephone interviews or personal interviews from January 2017 to December 2018. Design weights (WTS_M) were provided by the Statistics Canada to each individual in PUMF and were applied to derive meaningful estimates. The survey results were based on the self-reported data. The PUMF was generated without collecting any personally identifiable information from the participants, and was made freely accessible to the public without any charges. For privacy reasons, bootstrap weights are not available in CCHS PUMF. The PUMF from CCHS used in this study was accessed online through ODESI (Ontario Data Documentation, Extraction Service and Infrastructure) data portal via the University of Alberta Library.

### Study variables

Of the 113,290 participants in the CCHS 2017/2018, 82,508 who were 35 years and above were included in this study. The primary outcome was the presence of multimorbidity or not, which was defined as a report of at least two or more diseases from the list of eight chronic diseases, including asthma, arthritis, COPD, diabetes, heart disease, high blood pressure, mood disorder, and stroke. These chronic diseases have been previously considered in several studies on multimorbidity and are the most prevalent diseases in Canada [2,3,11]. To identify the presence of chronic diseases, each participant was asked *“We are interested in "long-term conditions" which are expected to last or have already lasted 6 months or more and that have been diagnosed by a health professional*. *Do you have <conditions>*?*”* The response to this question by the participants was used to determine the presence of each of the chronic diseases. A binary indicator variable was defined to indicate the presence of multimorbidity (two or more chronic diseases) with “1” for having multimorbidity and “0” for not having multimorbidity. Variables expected to have an association with multimorbidity were identified from previous studies on multimorbidity [2,3,5]. The body mass index (BMI) was determined by dividing the weight in kilograms by the square of the height in meters. The variables were categorized as follows: age (35–49, 50–64, 65+), sex (female or male), cultural background (white, non-white), obesity (underweight BMI < 18.5 kg/m^2^, normal weight 18.5kg/m^2^ ≤ BMI ≤ 25kg/m^2^, overweight 25kg/m^2^ < BMI ≤ 30kg/m^2^, obese BMI ≥ 30 kg/m^2^), marital status (single, widowed/divorced/separated, common-law/married), province [British Columbia, Prairies (Alberta, Saskatchewan, Manitoba), Ontario, Quebec, Atlantic (Newfoundland and Labrador, Nova Scotia, New Brunswick, Prince Edward Island), territories], household income (less than less than $39,999, $40,000 to $59,000, $60,000 to $79,999, $80,000 or more), highest household education (less than secondary school graduation education, secondary school graduation no post-secondary, post-secondary certificate diploma or university), smoking status (non-smoker, former smoker, current smoker), alcohol (no, occasionally, weekly), physical activity (inactive: PAADVVOL = 0, moderate: 0<PAADVVOL<900, active: PAADVVOL = > 900) and stress (not and not very stressful, somewhat, extreme). Physical activity level was categorized based on the guideline by the World Health Organization (WHO), which was derived by volume of weekly activity done in the past 7 days (PAADVVOL, unit: METs*minutes/week) [15]. A MET stands for a theoretical measurement that symbolizes the energy used during a physical endeavor. The extent of the effort is computed by multiplying the duration of the activity (at a specific intensity level) by the corresponding MET score linked to that level of intensity. Listwise deletion was used to exclude subjects with missing data for the outcome or independent variables in the logistic regression analysis.

### Statistical analysis

The design weights provided by Statistics Canada were used in all the analyses. The distribution of characteristics of the study sample was described using proportions, and the prevalence of multimorbidity was described for the total sample and within each category of the characteristics using proportions and 95% confidence intervals. The prevalence of each of the eight chronic diseases was stratified by sex and described with proportions and 95% confidence intervals. Survey-weighted univariate logistic regression was conducted to examine the association between sociodemographic characteristics and health behaviour risk factors and multimorbidity. The unadjusted odds ratios, 95% confidence intervals and level of significance were used to describe the strength of any association identified. All independent variables were initially included in the logistic regression and a purposeful selection method, instead of stepwise procedures, was used to determine the final model. During the model building, all independent variables were considered for inclusion in the logistic regression. To overcome the limitations of stepwise procedures, a purposeful selection method was used, which allowed for inclusion of biologically and clinically important variables in addition to statistically significant variables. This method involves a sequence of steps as outlined by Hosmer and Lemeshow [16]. Initially, the variables that are significant one at a time at less than 0.20 level of significance are identified, secondly these significant variables are included in a multivariable logistic regression and finally, the variables that are significant at a 0.05 level of significance, and relevant confounders, are kept in the final main effects model. The statistical significance of all plausible interactions was examined between each independent variables in the final main effects model. Only the first-order interaction was considered, and no high-level interactions were included in the analysis. The goodness of fit of the final model was evaluated by the Hosmer-Lemeshow test. Statistical analysis was performed using Stata version 15.0 and SAS version 9.4. A p-value of ≤ 0.05 was considered statistically significant.

## Results

**Fig 1:**
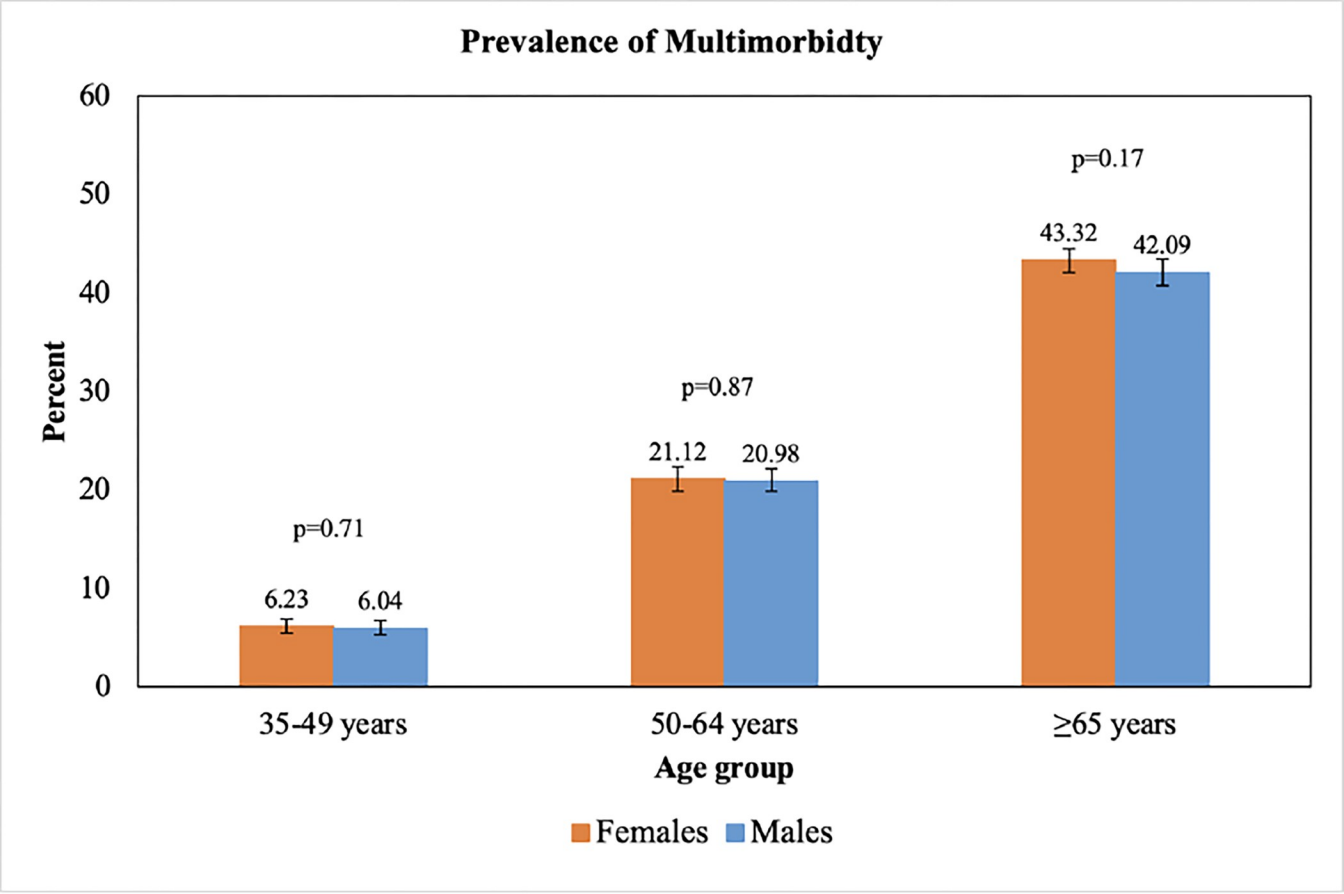
Prevalence of multimorbidity by age group and sex. Error bars indicate 95% confidence intervals.

As shown in Table 1, the prevalence of multimorbidity in the middle-aged and older adults was 22.20% (95% CI: 21.74%, 22.67%). Multimorbidity was significantly more prevalent in females than in males (22.82% vs. 21.55%, p = 0.01) which was consistent across all age groups (Fig 1).

**Table 1:** Characteristics of the study sample, distribution of multimorbidity by factors, and odds ratio for the association between sociodemographic and lifestyle characteristics and multimorbidity.

	% population		Prevalence for multimorbidity (%, 95% CI)	Unadjusted OR (95% CI)	*p-*value*
**Total sample**		82,508	22.20 (21.74, 22.67)		
**Sex**					0.01
** Female**		51.29	22.82 (22.18, 23.48)	1.00	
** Male**		48.71	21.55 (20.89, 22.21)	0.93 (0.88, 0.98)	
**Age group (years)**					<0.001
** 35–49**		34.50	6.14 (5.66, 6.65)	1.00	
** 50–64**		36.49	21.05 (20.54, 22.22)	4.08 (3.69, 4.50)	
** > = 65**		29.01	42.75 (41.88, 43.64)	11.41 (10.41, 12.53)	
**Culture background**					<0.001
** Non-white**		20.29	15.99 (14.68, 17.39)	1.00	
** White**		79.71	23.43 (22.95, 23.91)	1.60 (1.45, 1.78)	
**Body Mass Index**					<0.001
** Under weight**		0.80	25.36 (19.64, 32.08)	1.00	
** Normal weight**		30.81	13.64 (12.97, 14.33)	0.46 (0.33,0.65)	
** Overweight**		38.49	19.55 (18.81, 20.32)	0.72 (0.51, 1.00)	
** Obese**		29.89	31.23 (30.29, 32.20)	1.34 (0.96, 1.86)	
**Marital Status**					<0.001
** Single**		11.49	19.02 (17.84, 20.25)	1.00	
** Widowed/Divorced/Separated**		17.13	33.81 (32.76, 34.87)	2.17 (1.98, 2.38)	
** Common-law/Married**		71.37	19.93 (19.37, 20.49)	1.06 (0.97, 1.16)	
**Province**					<0.001
** British Columbia**		13.41	20.06 (18.96, 21.21)	1.00	
** Prairies**		16.87	22.05 (21.11, 23.01)	1.12 (1.03, 1.23)	
** Ontario**		23.82	20.74 (19.93, 21.58)	1.04 (0.96, 1.14)	
** Quebec**		38.63	22.74 (21.85, 23.65)	1.17 (1.07, 1.28)	
** Atlantic**		7.00	28.73 (27.54, 29.96)	1.60 (1.47, 1.76)	
** Territories**		0.27	20.16 (17.68, 22.89)	1.01 (0.84, 1.20)	
**Household income**					<0.001
** less than $39,999**		19.44	34.42 (33.36, 35.49)	1.00	
** $40,000 to $59,000**		14.78	27.32 (25.70, 28.15)	0.72 (0.66, 0.74)	
** $60,000 to $79,999**		12.79	23.99 (22.71, 25.33)	0.60 (0.55, 0.66)	
** $80,000 or more**		52.99	15.85 (15.25, 16.48)	0.36 (0.34, 0.38)	
** Highest household education**					<0.001
** Less than secondary** **school graduation education**		13.29	39.38 (37.97, 40.81)	1.00	
** Secondary school graduation,** **no post-secondary**		21.69	24.41 (23.41, 25.45)	0.50 (0.46, 0.54)	
** Post-secondary certificate** **diploma or university**		65.02	17.62 (17.09, 18.17)	0.33 (0.31, 0.35)	
**Smoking status**					<0.001
** Non-smoker**		51.36	18.36 (17.70, 19.03)	1.00	
** Former smoker**		32.51	28.03 (27.23, 28.85)	1.73 (1.63, 1.84)	
** Current smoker**		16.13	22.53 (21.43, 23.66)	1.29 (1.20, 1.40)	
**Alcohol**					<0.001
** No**		22.04	30.78 (29.58, 32.00)	1.00	
** Occasionally**		23.79	24.32 (23.32, 25.34)	0.72 (0.67, 0.78)	
** Weekly**		21.33	16.94 (16.11, 17.79)	0.46 (0.42, 0.50)	
** Daily**		32.84	18.27 (17.58, 18.98)	0.50 (0.47, 0.54)	
**Physical activity**					<0.001
** Inactive**		25.00	33.38 (32.29, 34.48)	1.00	
** Moderate**		38.12	20.97 (20.24, 21.72)	0.53 (0.50, 0.57)	
** Active**		36.87	15.25 (14.63, 15.88)	0.36 (0.34, 0.38)	
**Stress**					<0.001
** Not and not very stressful**		38.88	23.70 (22.98, 24.43)	1.00	
** Somewhat**		57.55	20.57 (19.96, 21.19)	0.83 (0.80, 0.88)	
** Extreme**		3.57	30.11 (27.25, 33.14)	1.39 (1.20, 1.60)	

*p value is from the univariate logistic regression.

The distribution of sociodemographic factors, proportion of multimorbidity by socio-demographic factors, and unadjusted odds ratios for the association of each factor with multimorbidity are shown in Table 1. Individuals in an older age group, of a white background, with obesity, who were not single, were living in any province except British Columbia, or were smoking, or having a stress level of ‘extreme’ were all more likely to have multimorbidity. In contrast, males, individuals with normal weight, individuals who reported higher household income, higher education level, drinking alcohol, undertaking physical activity, or having a stress level of ‘somewhat’ were all less likely to have multimorbidity.

As shown in Table 2, among the eight chronic diseases included in the definition of multimorbidity, the three most common chronic diseases were arthritis (26.67%), high blood pressure (25.35%), and diabetes (10.38%). When the distribution of chronic diseases was stratified by sex, females had a greater prevalence of arthritis (30.83% vs. 22.29%; p<0.001), mood disorder (10.96% vs. 6.76%; p<0.0001), asthma (9.24% vs. 6.18%; p<0.0001), and chronic obstructive pulmonary disease (4.39% vs. 3.98%; p = 0.03), whereas males had a greater prevalence of diabetes (12.09% vs. 8.87%; p<0.001) and heart disease (7.72% vs. 5.42%; p<0.001). A higher prevalence of high blood pressure was identified for males, and stroke was also observed in males more than females, although this last difference was not statistically significant.

**Table 2:** Prevalence of chronic diseases in the overall, male and female populations.

Chronic disease	Overall population Proportion (%, 95% CI)	Males Proportion (%, 95% CI)	Females Proportion (%, 95% CI)	*p-value* *
**Arthritis**	26.67 (26.16, 27.17)	22.29 (21.61, 22.98)	30.83 (30.10, 31.56)	<0.001
**High blood pressure**	25.35 (24.83, 25.87)	26.27 (25.51, 27.04)	24.47 (23.77, 25.18)	<0.001
**Diabetes**	10.38 (10.04, 10.74)	12.09 (11.55, 12.65)	8.87 (8.32, 9.23)	<0.001
**Mood disorder**	8.91 (8.58, 9.25)	6.76 (6.33, 7.20)	10.96 (10.47, 11.48)	<0.001
**Asthma**	7.75 (7.44, 8.07)	6.18 (5.79, 6.61)	9.24(8.78, 9.72)	<0.001
**Heart disease**	6.54 (6.29, 6.80)	7.72 (7.32, 8.14)	5.42 (5.11, 5.75)	<0.001
**COPD**	4.19 (4.01, 4.39)	3.98 (3.73, 4.26)	4.39 (4.14, 4.66)	0.03
**Stroke**	1.78 (1.64, 1.93)	1.90 (1.70, 2.12)	1.67 (1.48, 1.88)	0.12

*p-value indicates the significance of the differences in the prevalence between males and females.

The results of the final logistic regression model are shown in Table 3. Significant interactions were observed between age group and household income, and smoking habit and stress, and these were included in the final model. Subjects who were widowed, divorced, or separated were more likely to have multimorbidity in comparison to those who were single (p = 0.03). In comparison to subjects living in British Columbia, only those living in Quebec were significantly less likely to have multimorbidity (p = 0.02). Subjects with a household income of $80,000 or more, regardless of their age group, were less likely to have multimorbidity in comparison to those with a household income of less than $39,999. Interestingly, the protective effect of household income on having multimorbidity decreased with age, with the older age group having higher odds of multimorbidity than those for the other two age groups. Subjects who reported being extremely stressed were more likely to have multimorbidity, regardless of smoking status, in comparison to those who reported being stressed or not very stressed. Among current smokers, subjects who reported being extremely stressed had a three-fold increase in the odds of having multimorbidity compared with those who reported being stressed or not very stressed (adjusted OR = 3.84, 95% CI:2.67, 5.52). Among non-smokers and former smokers, a significant association was also observed between the level of stress and multimorbidity, with the odds of multimorbidity being lower than that observed for current smokers.

**Table 3:** Results from the logistic regression of multimorbidity for adults aged 35 and above allowing significant interactions.

Characteristics	Main effects +interactions model	
	Adjusted OR (95% CI)	*p-value*
**Total sample**		
**Sex**		
** Female**	1.00	
** Male**	1.04(0.98, 1.12)	0.21
**Age group (years)**		
** 35–49**	1.00	
** 50–64**	4.21(3.49, 5.08)	<0.001
** > = 65**	8.72(7.29,10.42)	<0.001
**Culture background**	-	
** Non-white**		
** White**		
**Obesity**		
** Underweight**	1.00	
** Normal weight**	0.57 (0.37, 0.89)	0.01
** Overweight**	0.91 (0.59, 1.42)	0.69
** Obese**	1.86 (1.19, 2.90)	0.01
**Marital Status**		
** Single**	1.00	
** Widowed/Divorced/Separated**	1.13 (1.01, 1.27)	0.03
** Common-law/Married**	0.99 (0.89, 1.10)	0.81
**Provinces**		
** British Columbia**	1.00	
** Prairies**	0.88 (0.70, 1.09)	0.24
** Ontario**	0.99 (0.80, 1.22)	0.92
** Quebec**	0.77 (0.62, 0.95)	0.02
** Atlantic**	0.95 (0.77, 1.18)	0.66
** Territories**	1.10 (0.89, 1.37)	0.38
**Highest household education**		
** Less than secondary school** **graduation education**	1.00	
** Secondary school graduation,** **no post-secondary**	0.80(0.73, 0.88)	<0.001
** Post-secondary certificate** **diploma or university**	0.75(0.69, 0.82)	<0.001
**Smoking status**		
** Non-smoker**	1.00	
** Former smoker**	1.35 (1.22, 1.49)	<0.001
** Current smoker**	1.19 (1.03, 1.39)	0.02
**Alcohol**		
** No**	1.00	
** Occasionally**	0.87 (0.79, 0.96)	0.01
** Weekly**	0.66 (0.60, 0.73)	<0.001
** Daily**	0.67 (0.61, 0.73)	<0.001
**Physical activity**		
** Inactive**	1.00	
** Moderate**	0.80 (0.74, 0.86)	<0.001
** Active**	0.64 (0.59, 0.70)	<0.001
**Household income** **(with people 35–49 years old)**		
** less than $39,999**	1.00	
** $40,000 to $59,000**	0.75 (0.56, 0.99)	0.05
** $60,000 to $79,999**	0.56 (0.40, 0.79)	0.001
** $80,000 or more**	0.55 (0.45, 0.68)	<0.001
**Household income** **(with people 50–64 years old)**		
** less than $39,999**	1.00	
** $40,000 to $59,000**	0.72 (0.60, 0.85)	<0.001
** $60,000 to $79,999**	0.65 (0.55, 0.77)	<0.001
** $80,000 or more**	0.51 (0.45, 0.59)	<0.001
**Household income** **(with people 65 years old+)**		
** less than $39,999**	1.00	
** $40,000 to $59,000**	0.96 (0.85, 1.07)	0.43
** $60,000 to $79,999**	0.93 (0.81, 1.06)	0.28
** $80,000 or more**	0.80 (0.72, 0.90)	<0.001
**Stress (with non-smokers)**		
** Not and not very stressful**	1.00	
** Somewhat**	1.34 (1.21, 1.50)	<0.001
** Extreme**	2.06 (1.53, 2.77)	<0.001
**Stress (with former smokers)**		
** Not and not very stressful**	1.00	
** Somewhat**	1.41 (1.28, 1.55)	<0.001
** Extreme**	1.75 (1.31, 2.33)	<0.001
**Stress (with current smokers)**		
** Not and not very stressful**	1.00	
** Somewhat**	1.64 (1.40, 1.93)	<0.001
** Extreme**	3.84 (2.67, 5.52)	<0.001

## Discussion

This study described the prevalence of multimorbidity, defined as having at least two or more chronic diseases concurrently among the middle-aged and older Canadian population based on cross-sectional data from the CCHS. In this study, the prevalence of multimorbidity was 22.20% in the Canadian population aged 35 years and above. Prior studies have used a variable definition of ‘middle-aged and older’ for estimation of the prevalence of multimorbidity. However, we found one newly published study based on 11,304 people from a 2018 National Health Service Survey in Yunnan, China which using a similar cut off and which reported a somewhat lower prevalence (10.12%) of multimorbidity, having two or more chronic conditions, among people aged 35 and above [17]. In another report based on the Canadian Chronic Disease Surveillance System, the prevalence of multimorbidity was 26.5%, slightly higher than the 22.20% reported here, and in an older population aged 40 years and above [5]. Many prior studies either included data from only one province, included differing proportions of older participants than this study, or identified fewer chronic diseases, all of which could contribute to the variability seen in the reported prevalence of multimorbidity across studies. Study population and data sources are other possible reasons that could contribute to the difference in the reported prevalence of multimorbidity.

Similar to the results observed in this study, a number of previous studies have suggested females were at greater risk of multimorbidity than males [2,11]. We also found that subjects who were widowed, divorced, or separated had higher odds of multimorbidity compared to those who were single. A recent longitudinal study based on multiple countries examining the martial status and prevalence of multimorbidity showed that the likelihood of multimorbidity increased with widowhood, which is in line with our study results [18]. Increased mental stress caused by losing a partner through death, divorce, or separation has been shown to have worse health outcome (e.g., increased number of physical diseases), and could be one of the possible explanations for the increase of multimorbidity in subjects who are widowed, divorced, or separated in this study [19,20]. However, the association between marital status and multimorbidity has remained controversial. A study of community-dwelling older adults aged 65 and older in Manitoba, Canada that showed no association between marital status and the prevalence of multimorbidity [10].

Previous research has given little attention to the association between lifestyle factors and multimorbidity. In this study, subjects who drank occasionally, weekly, or daily were less likely to have multimorbidity in comparison to non-drinkers, which was similar to the findings from the cross-sectional analysis of the Canadian Longitudinal Study on Aging [12]. Some studies have shown that moderate levels of alcohol consumption were likely to reduce the risk of the development of some chronic diseases such as dementia and stroke [21]. The reasons for the inverse association between alcohol intake and multimorbidity are unknown but may be related to selection bias, with subjects having conditions associated with drinking being less likely to participate in the study than healthy individuals, or possibly subjects with multimorbidity stopping alcohol use because of advice from their health care providers [22]. In this study, participants who were physically active had lower odds of multimorbidity in comparison to those who were inactive. Similar findings were found in a cross-sectional study of the Canadian population aged 20 years and older, which also showed that being inactive was associated with a higher prevalence of multimorbidity [2]. An inverse relationship between multimorbidity and physical activity for both younger and older participants has been reported [23]. In this study, individuals with the highest education levels had lower odds of multimorbidity in comparison to those with the lowest education levels. Similar findings were found in Scottish patients, which argued that there was a strong graded association between social position and multimorbidity [24]. A recent meta-analysis also showed that lower education levels were associated with a 64% increase in odds of multimorbidity in comparison to higher education levels [25].

A number of important interactions were identified in this study. Firstly, we found a significant interaction between income and age. Canada has a universal health care system which provides free health care to all its citizens and differences in the age-based health coverage or insurance could not be considered as a possible reason for the interaction between income and age on multimorbidity. Higher household income had a protective effect on multimorbidity in all age groups, but the protective effect appeared less strong in the older age group. Similar results were also demonstrated of a ‘buffering’ effect of age on the positive relationship between household income and the average number of conditions comprising multimorbidity, especially in patients aged 65 and older, in other reports [12]. The association between older age and multimorbidity could be a plausible explanation for the reduced protective effect of household income on multimorbidity. This association is extremely strong that it could potentially overshadow the effect of other contributing factors, in this case, household income. It is important to note that although the effect of household income on multimorbidity decreased with age, the absolute prevalence of multimorbidity was significantly higher among elderly patients in comparison to their younger counterparts in all income groups. The significant interaction between income and age suggests that addressing income disparities is crucial for reducing the risk of multimorbidity. Further public health policies should focus on narrowing socioeconomic gaps to ensure equitable access to healthcare resources. Moreover, it may be beneficial to develop targeted interventions for middle-aged patients as they are most likely to be affected by the effect of household income on multimorbidity.

Another significant interaction, between stress and smoking status, was also identified. While those who reported having extreme stress had a higher risk of multimorbidity than those who reported being not very stressed regardless of smoking status, a stronger association between stress and multimorbidity was observed among current smokers. Although an interaction between stress and smoking among patients with multimorbidity has not been reported in previous studies, it has been shown that higher level of stress was significantly associated with having more than three chronic conditions [26] and smoking has been shown to be pre-disposing factor for multimorbidity in initially disease-free population in a cohort study from Finland [27]. A cross-sectional study based on patients with multimorbidity aged 50 years and older living in six low- and middle-income countries showed that patients with higher levels of multimorbidity were more likely to report higher levels of stress than those without multimorbidity [28]. It is also well-known that smoking is associated with the development of chronic diseases such as cardiovascular disease [29]. Thus, it has been shown in the previous studies that both smoking and stress are independent risk factors for multimorbidity. Our finding of a stronger association between stress and multimorbidity among both former and current smokers is potentially interesting but needs further elucidation. Smokers may be at risk of multimorbidity due to a direct influence of smoking, and smoking may also be used by some as a mechanism for managing a greater stress level. A prospective study of Danish adults found that increased stress was associated with a higher likelihood of continuing to smoke, supporting the notion that the relationship between smoking and stress is not simple [30]. Thus, it is possible to argue that smoking status may mediate the relationship between stress and multimorbidity. However, given the nature of a cross-sectional study, such as this, it is impossible to ascertain causality. Therefore, one must exercise caution when interpreting the interaction between smoking status and perceived stress in patients with multimorbidity. Future longitudinal studies are required to have a better understanding of the underlying directionality of the relationship between smoking status, perceived stress and multimorbidity.

Our study has a number of strengths and limitations. Firstly, the data used for this study were obtained from a national population-based health survey utilizing validated survey designs. Therefore, the results were representative of the overall Canadian population. Secondly, two plausible interactions were identified in the study which has provided a better understanding of factors associated with multimorbidity. Unlike a number of prior studies, this study included mental health-related problems in the definition of multimorbidity, which was suggested by the 2019 Canadian Chronic Disease Indicators report [31]. Due to the nature of cross-sectional study design, causal relationships cannot be established from the results from this study. A limited number of independent variables was considered in the multivariable logistic model, therefore, residual confounding is likely to exist in at least some of the reported results. The self-reported clinical diagnosis of chronic disease in this study potentially makes the results prone to recall bias. Moreover, differential in access to healthcare providers may result in under diagnosis of chronic diseases in some parts of Canada with consequent potential for causing errors and biases in the findings in this study. A stepwise selection was used to identify the interactions between the variables in the final model. Significant interactions between the variables were identified one at a time and were included in the logistic regression, and non-significant interactions were then removed from the final model. It is possible that using this stepwise approach might result in missing some biologically important or clinically important interactions.

## Conclusions

Our study found that the prevalence of multimorbidity in the middle-aged and older Canadian population was greater in females than males, and was increased among those with obesity, those with no alcohol intake, those who were inactive, those with lower education levels, or who were widowed, divorced or separated, and was decreased among those living in Quebec. The relative protective effect of household income on having multimorbidity decreased with aging. Current smokers who reported being extremely stressed had an increased likelihood of multimorbidity. The findings in this study illustrate the importance of examining the interactions between risk factors rather than considering them only independently. The outcomes of our study will enhance comprehension regarding the determinants associated with multimorbidity, facilitating the identification of sub-populations that were most vulnerable to developing multimorbidity. A deep understanding of underlying mechanisms is necessary among patients living in low and middle household income to allow better prevention and management of multimorbidity. Future longitudinal studies are required to provide causal evidence.
